# Promotional Effects on the Catalytic Activity of Co-Fe Alloy Supported on Graphitic Carbon for CO_2_ Hydrogenation

**DOI:** 10.3390/nano12183220

**Published:** 2022-09-16

**Authors:** Bogdan Jurca, Lu Peng, Ana Primo, Alvaro Gordillo, Amarajothi Dhakshinamoorthy, Vasile I. Parvulescu, Hermenegildo García

**Affiliations:** 1 Department of Organic Chemistry and Biochemistry and Catalysis, Faculty of Chemistry, University of Bucharest, Bdul Regina Elisabeta 4-12, 030016 Bucharest, Romania; 2 Instituto Universitario de Tecnología Química, Universitat Politècnica de València-Consejo Superior de Investigaciones Científicas, Av. De los Naranjos s/n, 46022 Valencia, Spain; 3 BASF SE, 67056 Ludwigshafen am Rhein, Germany; 4 Departamento de Química, Universitat Politècnica de València, Av. De los Naranjos s/n, 46022 Valencia, Spain; 5 School of Chemistry, Madurai Kamaraj University, Madurai 625021, Tamil Nadu, India

**Keywords:** graphitic carbon, CO_2_ methanation, alloy nanoparticles

## Abstract

Starting from the reported activity of Co-Fe nanoparticles wrapped onto graphitic carbon (Co-Fe@C) as CO_2_ hydrogenation catalysts, the present article studies the influence of a series of metallic (Pd, Ce, Ca, Ca, and Ce) and non-metallic (S in various percentages and S and alkali metals) elements as Co-Fe@C promoters. Pd at 0.5 wt % somewhat enhances CO_2_ conversion and CH_4_ selectivity, probably due to H_2_ activation and spillover on Co-Fe. At similar concentrations, Ce does not influence CO_2_ conversion but does diminish CO selectivity. A 25 wt % Fe excess increases the Fe-Co particle size and has a detrimental effect due to this large particle size. The presence of 25 wt % of Ca increases the CO_2_ conversion and CH_4_ selectivity remarkably, the effect being attributable to the CO_2_ adsorption capacity and basicity of Ca. Sulfur at a concentration of 2.1% or higher acts as a strong poison, decreasing CO_2_ conversion and shifting selectivity to CO. The combination of S and alkali metals as promoters maintain the CO selectivity of S but notably increase the CO_2_ conversion. Overall, this study shows how promoters and poisons can alter the catalytic activity of Co/Fe@C catalysts, changing from CH_4_ to CO. It is expected that further modulation of the activity of Co/Fe@C catalysts can serve to drive the activity and selectivity of these materials to any CO_2_ hydrogenation products that are wanted.

## 1. Introduction

Within the context of decreasing CO_2_ emissions into the atmosphere and the valorization of this gas as feedstock, there is an increasing interest in developing efficient catalysts for CO_2_ hydrogenation to hydrocarbons [1,2,3,4,5,6,7,8,9,10,11]. In a series of papers, we have been reporting that Co-Fe alloy nanoparticles (NPs) wrapped onto defective N-doped graphene [Co-Fe@(N)C] are suitable catalysts that, by fine-tuning, can exhibit selectivity toward the formation of methane [12], the reverse water gas shift [13], or the formation of a significant percentage of C_2+_ products [14]. It seems that, in these types of catalysts, particle size is one key parameter that determines product selectivity.

Continuing with this line of research, it is well known that the presence of certain elements, generally known as promoters, can determine the performance of catalysts in CO_2_ and CO hydrogenation [15,16,17,18,19,20,21,22,23,24,25,26,27]. Specifically, alkali metals, such as K and Cs, can alter the activity of hydrogenation catalysts by altering the adsorption properties and surface basicity, among other effects [20,25,28,29,30]. In this context, it would be of interest to establish what effects the different promoters have on the catalytic activity of Co-Fe alloy NPs wrapped onto graphitic carbon, with the long-term goal of finding even more active, selective, and stable catalysts.

In recent years, the use of graphene-based solids as catalysts for CO_2_ hydrogenation has attracted much attention [31]. For example, a Pd-embedded g-C_3_N_4_/reduced graphene oxide (rGO) aerogel (Pd-g-C_3_N_4_/RGOA) photocatalyst was reported for the reduction of CO_2_ to CH_4_ [32]. In another precedent, the influence of nickel over rGO was studied in the hydrogenation of CO_2_ to CH_4_ [33]. Furthermore, a three-dimensional cobalt nanocrystal supported over rGO was also tested for the CO_2_ hydrogenation reaction [34]. Iron-based catalysts with honeycomb-structured graphene as the support and potassium as the promoter were also employed as catalysts for the direct hydrogenation of CO_2_ to light olefins [35].

With this objective in mind, the present study focuses on the influence that metallic (Na, K, Ca, Pd and Ce) and non-metallic (S) promoters exert on the performance of Co-Fe alloy NPs wrapped around graphitic carbon, either with N-doping [Co-Fe@(N)C] or without N-doping (Co-Fe@C) as CO_2_ hydrogenation catalysts. The promoters and their concentrations were selected based on precedents reporting the influence of alkali [36], alkali-earth [37,38], and noble metals [39,40,41] on hydrogenation catalysts, as well as the poisoning effect of S on the Fischer-Tropsch catalysts [42,43,44]. Evidence will be presented showing a significant influence of the promoters on the performance of Co-Fe@(N)C and Co-Fe@C catalysts, driving the selectivity toward CH_4_ or the reverse water gas shift.

## 2. Materials and Methods

### Synthesis of Co-Fe@(N)C and Co-Fe@C

Commercially available reagents were purchased from Sigma Aldrich (Merck KGaA, Darmstadt, Germany) and used without further purification.

Route (a): Samples 1 and 2 were prepared following route (a). Briefly, 1000 mg chitosan and 625 μL acetic acid were added into 50 mL milli-Q water. After the chitosan dissolved completely, the solution was introduced dropwise with a syringe (0.8 mm diameter needle) into an aqueous solution of sodium hydroxide (500 mL; 2M). The gel microspheres were formed and immersed in NaOH solution for 2 h, then profusely washed with distilled water until the pH = 7. Then the resulting hydrogel microspheres were washed in a series of ethanol/water baths with an increasing concentration of ethanol (10, 30, 50, 70, 90, and 100 volume percent, respectively) for 15 min in each bath and then immersed in 100 mL Co-Fe-ethanol or Co-Fe-Pd-ethanol solution with different concentrations for 2d with slow stirring. After that, the microspheres were reduced with 375 mL 0.05 M NaBH_4_-ethanol solution for 5 h, after which it was exchanged for supercritical CO_2_. The resulting microspheres were pyrolyzed under an Ar flow (200 mL/min), increasing the temperature at a rate of 2 °C/min up to 200 °C for 2 h, then increasing the temperature to 900 °C for 2 h. The preparation of samples 8–11 was performed following route (a) with an additional thiourea impregnation (375 mL) step in EtOH after the NaBH_4_ reduction and before supercritical CO_2_ drying and pyrolysis.

Route (b): Samples 3 and 4 were prepared following route (b). Briefly, 1000 mg chitosan, 625 μL acetic acid, and a certain amount of Co(OAc)_2_ and Fe(OAc)_2_ or Ce(OAc)_3_·xH_2_O were added to 50 mL milli-Q water. After the chitosan dissolved completely, the solution was introduced dropwise with a syringe (0.8 mm diameter needle) into an aqueous solution of sodium hydroxide (500 mL; 0.1M). The gel microspheres were formed and then immersed in NaOH solution for 1 h, then profusely washed with distilled water to attain a pH = 7. Then the resulting hydrogel microspheres were washed in a series of ethanol/water baths with an increasing concentration of ethanol (10, 30, 50, 70, 90, and 100 volume percent, respectively) for 15 min in each bath. After that, the microspheres were reduced with 500 mL NaBH_4_-ethanol solution (0.05M) overnight and exchanged for supercritical CO_2_. The resulting microspheres were pyrolyzed under an Ar flow (200 mL/min), increasing the temperature at a rate of 2 °C/min, then increasing to 200 °C for 2 h, and finally increasing to 900 °C for 2 h.

Route (c): Samples 5–7 were prepared following route (c). Following the method for samples 5 and 7, 1000 mg sodium alginate was added to 50 mL milli-Q water. After the sodium alginate dissolved completely, the solution was introduced dropwise, with a syringe (0.8 mm diameter needle), into 100 mL of an aqueous solution of CoCl_2_·6H_2_O and FeCl_2_ or Ce(OAc)_3_·xH_2_O. The gel microspheres were formed and immersed in the solution overnight. Then, the resulting hydrogel microspheres were washed in a series of ethanol/water baths with an increasing concentration of ethanol (10, 30, 50, 70, 90, and 100 volume percent, respectively) for 15 min in each bath and then exchanged for supercritical CO_2_. Unlike samples 5 and 7, sample 6 was prepared by precipitating alginate acid aqueous solution (30 mL; 2 g alginic acid; 2.5 ml ammonia) into the CaCl_2_ aqueous solution (4 g; 100 mL), then profusely washed with distilled water. Then, the resulting hydrogel microspheres were washed in a series of ethanol/water baths with an increasing concentration of ethanol (10, 30, 50, 70, 90 and 100 volume percent, respectively) for 15 min in each bath. Afterward, the alcogel microspheres were immersed in Fe-Co-ethanol solution for 1 day, then washed with anhydrous ethanol and exchanged for supercritical CO_2_. The resulting microspheres were pyrolyzed under Ar flow (200 mL/min), increasing the temperature at a rate of 2 °C/min up to 200 °C for 2 h and then to 900 °C for 2 h.

The detailed procedures of catalyst characterization and catalytic testing are reported in the Supplementary Materials.

## 3. Results

**Scheme 1:**
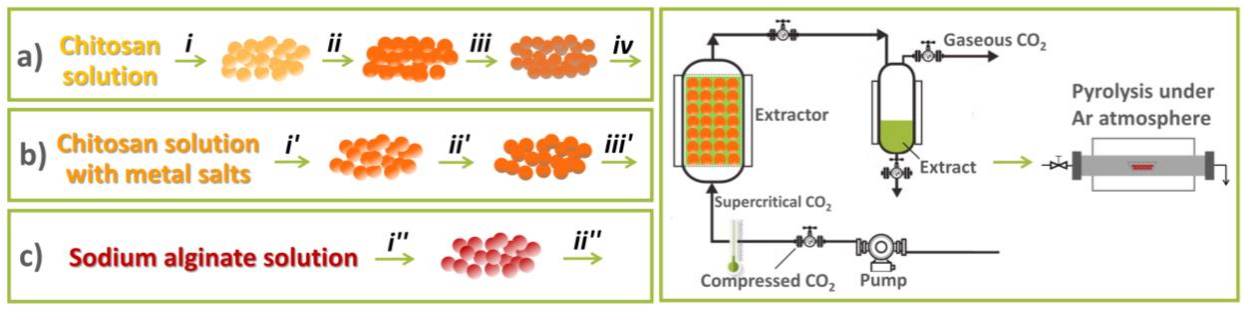
Procedure used to prepare the Co-Fe@(N)C (routes (**a**,**b**)) and Co-Fe@C (route (**c**)) under study. Preparation of samples 1, 2, and 8–11**,** (***i***) precipitation in NaOH solution; (***ii***) water/ethanol exchange and metal salt impregnation; (***iii***) NaBH_4_ reduction in ethanol solution; (***iv***) supercritical CO_2_ drying. (Samples 8–11 were created following route (**a**) with an additional thiourea impregnation in ethanol in step (***iii***). Samples 3 and 4 (***i’***) were created by precipitation in NaOH solution; (***ii’***) water/ethanol exchange and NaBH_4_ reduction in ethanol solution; (***iii’***) supercritical CO_2_ drying. Sample 5–7, (***i’’***) precipitation in metal aqueous solution and water/ethanol exchange; (***ii’’***) supercritical CO_2_ drying.

The series of Co-Fe@(N)C and Co-Fe@C catalysts modified with the promoters under study are listed in Table 1, which also includes the relevant analytical data for these samples. Samples 1, 3, and 5 have been previously reported by us in previous studies [12,14]. The other samples appearing in Table 1 serve to determine the influence of promoters (Pd, Ce, Ca) or poison (S) on their activity and selectivity. The reader is referred to the published literature for a more extensive characterization of samples 1, 3, and 5 [12,13,14]. These materials were prepared following three different routes. The need for different routes in the preparation of the series of materials was derived from the use of two different graphitic carbon precursors, chitosan/alginate, and the need to have better control of particle size distribution. In two of the routes, the carbon precursor was chitosan, rendering N-doped graphitic carbon [Co-Fe@(N)C], while alginate was the precursor in the third route for the preparation of Co-Fe alloy NPs on defective graphitic carbons that do not contain the N element (Co-Fe@C). Chitosan, being a polymer of glucosamine and with a 6.25 wt % of Nm acts in the pyrolysis as a simultaneous source of C and N, while alginate is a copolymer of D-manuronic and L-guluronic acids, condensed through a glycosidic β-(1,4) bond, and does not contain N in its composition. Scheme 1 illustrates the three preparation procedures.

In route (a), chitosan microspheres of millimetric size were first obtained as hydrogel by the precipitation of chitosan dissolved in acid water into a strong basic NaOH aqueous solution. The resulting chitosan hydrogel was converted into alcogel by a gradual exchange of H_2_O with EtOH. Then, Co^2+^ and Fe^2+^ salts were adsorbed onto chitosan beads in EtOH, before chemical reduction with NaBH_4_ and subsequent supercritical CO_2_ drying, followed by pyrolysis. It has been previously observed that NaBH_4_ reduction of Co^2+^ and Fe^2+^ adsorbed on chitosan renders after the pyrolysis of Co-Fe alloy NPs of narrow particle distribution, in which the average particle dimension can be controlled to a certain extent in the range from 8 to 17 nm [14].

Route (b) adsorbs Co^2+^ and Fe^2+^ salts in an acid aqueous chitosan solution, before the formation of the millimetric beads and the exchange of H_2_O by EtOH, supercritical CO_2_ drying, and pyrolysis. While this route does not use a chemical reducing agent, the particle size distribution of the Co-Fe alloy NPs tends to be broader than in route (a) [12]. It should be mentioned that according to prior results, a broader particle size distribution of the Co-Fe alloy NPs tends to favor the formation of CH_4_ as the prevalent product [12].

The precursor of the carbon residue in route i was sodium alginate, which adsorbs Co^2+^ and Fe^2+^ in an aqueous solution and is then precipitated with a concentrated solution of divalent metals, either Fe^2+^ in excess (sample 5) or Ca^2+^ (samples 6 and 7) in H_2_O. Alginate is soluble in aqueous solutions at pH values higher than 5, but it is not soluble in the presence of an excess of di- and tri-positive cations, due to the crosslinking of the linear alginate fibrils [45]. The process is completed by the conversion of alginate hydrogel into alcogel by the gradual exchange of H_2_O with EtOH, followed by supercritical CO_2_ drying and pyrolysis at 900 °C. The main difference between routes (i) and (a) and (b) is seen in the different solubilities of chitosan and alginate in acid and neutral-basic aqueous solutions, respectively [46].

As can be deduced from Table 1, the set of samples was prepared with the objective of determining the possible influence as promoters of Pd (samples 1 and 2), Ce^3+^/^4+^ (samples 3 and 4), an excess of Fe^2+^ (sample 5), Ca^2+^ (sample 6), the combination of Ca^2+^ and Ce^3+^/^4+^ (sample 7) or Na^+^ and K^+^, in combination with S (sample 11), on catalytic activity. The percentage of Co, Fe, and metallic promoters was determined by ICP-OES of the liquors after the digestion of the samples in aqua regia. In all the cases, except Fe^2+^ and Ca^2+^, the percentage of the promoter was purposely low, under 0.5 wt %. The special cases were Fe^2+^ and Ca^2+^ as promoters. Since the alginate beads were precipitated by Fe^2+^ or Ca^2+^, the content of this alkali-earth metal was much higher, about 25 wt %, compared to the other promoters under study.

Based on the precedents regarding the influence of S in hydrogenation catalysts, increasing the product selectivity by decreasing the catalytic activity, an additional set of four Co-Fe@(N)C samples was prepared containing this element. The S content, as well as the percentage of C and N, were quantified by elemental combustion analyses. The relevant analytical details of samples 8–11 are also included in Table 1. The preparation of samples 8–11 was performed following route (a), with an additional thiourea impregnation step in EtOH after the NaBH_4_ reduction of Co^2+^ and Fe^2+^ salts and before supercritical CO_2_ drying and pyrolysis.

**Figure 1:**
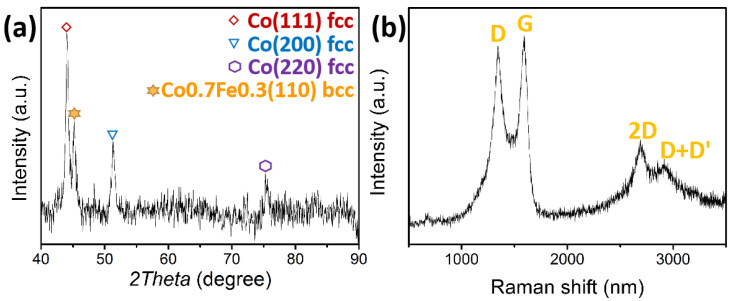
XRD pattern (**a**) and Raman spectrum (**b**) of sample 1.

Promoter-containing Co-Fe@(N)C and Co-Fe@C samples were characterized by powder XRD, Raman spectroscopy and electron microscopy. As expected, in view of the related precedents in the literature, the XRD patterns indicate that during the pyrolysis, Co and Fe became reduced into the metallic state, with the metal NPs having a variable proportion of fcc and bcc phases. Figure 1 presents a selected XRD pattern for sample 1, while the full set of XRD patterns is gathered in Figure S1 of the Supplementary Materials. Importantly, the comparison of the XRD pattern of sample 1, lacking the promoter, with those of the rest of the samples in which promoters in low amounts were present did not reveal any difference in the XRD pattern, except in the case of sample 6, which was characterized by a high Ca content. This lack of influence of promoters on the XRD spectra of the Co-Fe@(N)C and Co-Fe@C samples can be attributed in general to the low percentage of promoters and their high dispersion. For sample 5, containing a large percentage of Fe in its composition, bcc was the prevalent phase. Additionally, in the case of sample 3, the Co/Fe bcc phase prevailed, probably due to its preparation method. Similarly, for S-doped samples, no additional diffraction peaks due to the S species could be identified in the XRD of samples 8–11. Only in the case of sample 6 was the presence of CaCO_3_ recorded, as characterized by their diffraction peaks at 39.4°, 47.5°, and 56.5°. The formation of CaCO_3_ can be understood by considering the ambient exposure of the samples after preparation and the prompt carbonation of CaO. As in the reported precedents [12,14], the distinction by XRD between independent Co and Fe phases with Co-Fe alloy is uncertain, due to the similarity of the unit cell parameters of Co and Fe.

The graphitic nature of the carbon residue was determined by Raman spectroscopy, in which the characteristic D + D’, 2D, G, and D peaks were recorded, appearing at 2960, 2700, 1590, and 1350 cm^−1^, respectively. As an example, Figure 1 also includes the Raman spectrum of sample 1, while Figure S2 in the Supplementary Materials collects the Raman spectra of all the samples under study. The intensity of the G vs. the D band (I_G_/I_D_) is generally taken as a quantitative indicator of the density of the defects [47]. In the present case, the I_G_/I_D_ ratio was between 1.15 and 1.25, which is common for the type of graphitic carbon obtained by the pyrolysis of chitosan or alginate [48]. The intensity of the overtones is also taken as a sign that the carbon residue is constituted by the stacking of only a few graphene layers, these overtones in the region between 2950 and 2700 cm^−1^ being apparent in most of the samples. These Raman spectra essentially coincide with those previously reported for the Co-Fe@(N)C samples lacking promoters [12,13,14]. This observation suggests that the promoters do not change the graphitic nature of the carbon residue formed in the pyrolysis process.

**Figure 2:**
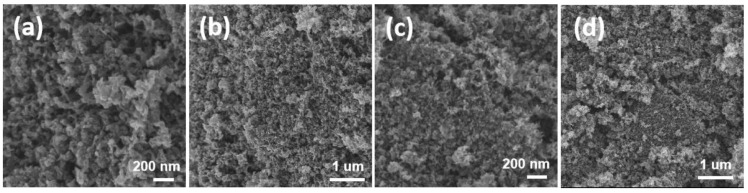
FESEM images of samples 1 and 6 (**a**,**b**): sample 1 and (**c**,**d**): sample 6.

The morphology of the Co-Fe@(N)C and Co-Fe@C samples containing promoters was imaged by field emission scanning electron microscopy (FESEM). Figure 2 presents some representative images, while a complete set of images of the Co-Fe@(N)C and Co-Fe@C materials is included in Figure S3 of the Supplementary Materials. It was observed that samples 1–11 are characterized by a highly spongy, fluffy structure that derives from the carbonization of the polysaccharide fibrils of the chitosan or alginate after supercritical drying [49]. It has been reported in the literature that in contrast to the behavior of hydrogels that give compact beads, the conversion of chitosan or alginate microspheres into alcogels and subsequent supercritical drying results in a highly porous, spongy, large surface area with beads of chitosan and alginate [49]. This different behavior is due to the occurrence in dry hydrogels of fibril close packing, derived from the formation of hydrogen bridges, while supercritical CO_2_ drying diminishes this fibril interaction considerably. Interestingly, with the resolution of the FESEM images, the presence of Co-Fe NPs was undetectable and no evidence of the presence of promoters on particle morphology could be obtained, even for CaCO_3_, which is present at a large percentage.

**Figure 3:**
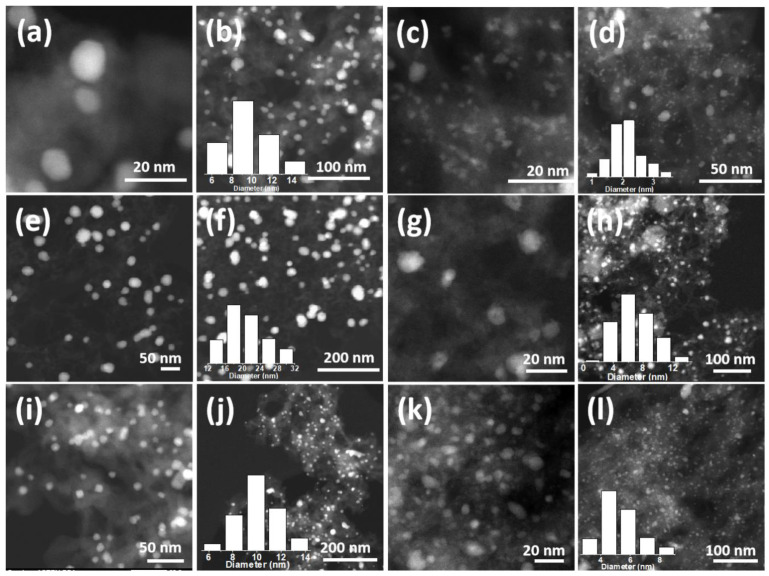
DF-TEM images (**a**-**l**) of samples 2, 4, 5, 6, 8, 11. (**a**,**b**): sample 2; (**c**,**d**): sample 4; (**e**,**f**): sample 5; (**g**,**h**): sample 6; (**i**,**j**): sample 8; (**k**,**l**): sample 11. Inset: the statistical particle size distributions of samples 2, 4, 5, 6, 8, and 11, respectively.

Transmission electron microscopy (TEM) images revealed the presence of Co-Fe NPs. Dark-field images allowed us to estimate the particle size distribution for samples, based on the measurement of the dimensions of a statistically relevant number of these particles. Figure 3 shows some representative images of the samples under study with the corresponding particle-size histograms, while a collection of additional images and size distribution measurements are provided in Figure S4 of the Supplementary Materials. Table 1 summarizes the average particle size for some samples. As can be seen, most of the samples exhibit a similar average particle size of about 10 nm, except sample 5, in which the particle size was significantly larger, at about 18 nm. This larger particle size of sample 5 can be easily explained, considering the much higher Fe content of this sample.

High-resolution TEM images allowed us to determine the interplanar distance of the 110 plane in Co-Fe NPs as 0.21 nm, which corresponds to the alloy between the two metals [13]. These images also reveal that the Co-Fe NPs are partially covered by one to three layers of defective graphene. The same characterization has been reported in the literature for similar Co-Fe@(N)C samples [12,13,14]. While the presence of promoters in some cases was not apparent from the TEM images, due to their low percentages, EDX analysis revealed the presence of the expected elements in the images. In the case of Ca as a promoter, it was observed that this element was coating the Co-Fe NPs as determined by analysis of the variation of the elemental composition along Co-Fe NPs in high-resolution TEM. Therefore, TEM characterization shows that promoters are well dispersed in the Co-Fe@(N)C and Co-Fe@C samples, with interaction with the Co-Fe alloy NPs supported on defective few-layer graphene layers.

### Catalytic Activity

The catalytic activity of the samples was evaluated under the continuous flow of a CO_2_-H_2_ mixture diluted in Ar in a pressurized tubular, stainless steel reactor, with the catalyst as a fixed bed. No binders were used, and the samples were used as fine powders. Each catalyst was tested in the range of temperatures from 300 to 500 °C, rising by 50 °C increments without removing the sample from the reactor. After setting a new temperature, the reactor was allowed to equilibrate, then the temperature was maintained for 1 h dwell time. The composition of the reaction mixture was determined by gas chromatography analysis at 30, 45, and 55 min after the temperature of the reaction was equilibrated. No differences larger than 10% among the three analyses were measured in most of the cases, and conversion and selectivity values for the temperature were taken as the average of the three independent analyses. For the few cases in which larger differences among the values were found, the measurement was not considered. 

Preliminary controls at the highest reaction temperature of the study in the absence of any catalysts, or when using 40 mg of (N)G or G without metals as catalysts, showed low CO_2_ conversions of 6, 13, and 8%, respectively, CH_4_ being the only detectable product. It has been previously reported that defective graphenes exhibit some activity as CO_2_ hydrogenation catalysts [50]. However, as previously found [12,13,14], these CO_2_ conversion values of (N)G and G are much lower under the conditions of the present study than those found when Co-Fe NPs were present in the catalyst.

All the Co-Fe@(N)C and Co-Fe@C samples containing promoters were active as catalysts for CO_2_ hydrogenation. The products observed were CH_4_, CO, and variable proportions of C_2_–C_4_ hydrocarbons (C_2_-C_4_^0^), including a certain proportion of alkenes (C_2-_C_4_^=^). While any product derived from CO_2_ hydrogenation is desirable, the key point is to develop a selective catalyst that can afford very high selectivity for a given product at very high CO_2_ conversion. As expected, CO_2_ conversion increased with the temperature, and selectivity varied in each case with the conversion. Differences in the catalytic activity of the Co-Fe@(N)C and Co-Fe@C samples that were attributable to the effect of promoters were observed.

**Figure 4:**
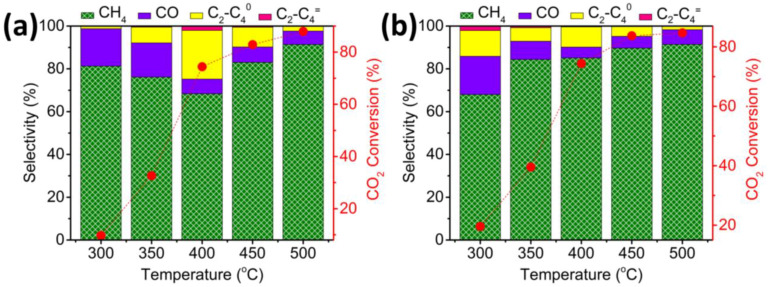
CO_2_ conversion and selectivity for samples 1 and 2 at different temperatures. (**a**): Sample 1 and (**b**): sample 2. Reaction condition: H_2_/CO_2_ ratio of 7, total flow 4 mL/min, 10 bar, 40 mg catalyst. The error of the measurements is lower than 10%.

The comparison of the catalytic activity of samples 1 and 2 shows that the presence of Pd in 0.5 wt % increases CO_2_ conversion in the lower temperature range from 300 to 400 °C, with some changes in selectivity. The difference in the catalytic performance of samples 1 and 2 is presented in Figure 4 and Table S2 and S3 in the Supplementary Materials. This change was particularly notable at 400 °C, the presence of Pd increasing CH_4_ selectivity. This effect can be explained by considering that Pd is a better hydrogenating metal than the Co-Fe alloy and it can activate H_2_ at lower temperatures. Subsequently, the H atoms on Pd would undergo spillover from the Fe-Co NPs. Since CH_4_ is the most stable hydrogenation product, the higher catalytic activity caused by Pd as a promoter would be reflected in higher CH_4_ selectivity.

Less evident is the case of Ce as a promoter (see Figure S5 and Table S4 and S5 in the Supplementary Materials). While a comparison of the catalytic activity of Co-Fe@(N)C samples 3 and 4 shows that Ce does not alter CO_2_ conversion significantly, CO selectivity was considerably reduced at every temperature, favoring the formation of CH_4_. In this sense, the effect on product selectivity by the promotion of Ce is analogous to that observed for Pd. In contrast, in the case of Co-Fe@C samples derived from calcium alginate, the presence of Ce in a small percentage has a detrimental effect, decreasing CO_2_ conversion substantially and resulting in mixtures with a large percentage of CO. This contrasting behavior could indicate that the role of Ce is not H_2_ activation, as is the case with Pd, but rather an interaction with Co-Fe with the tuning of their acidity. Therefore, since CaCO_3_ should essentially have the role of the base, the promotional effect of Ce would be different in samples 4 and 7.

**Figure 5:**
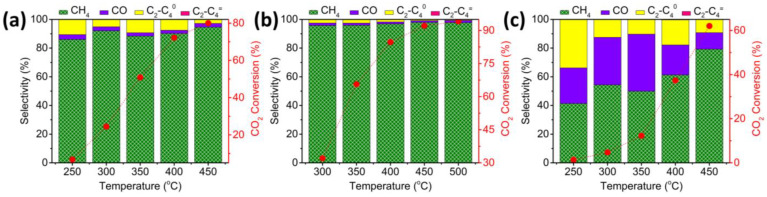
CO_2_ conversion and selectivity for samples 5–7 at different temperatures. (**a**): sample 5; (**b**): sample 6 and (**c**): sample 7. Reaction condition: H_2_/CO_2_ ratio of 7, total flow 4 mL/min, 10 bar, 40 mg catalyst.). The error of the measurements is lower than 10%.

As commented earlier, an excess of Fe^2+^ or Ca^2+^ was employed in procedure C to form insoluble alginate beads, and these two metals are present in much higher weight percentages in samples 5–7. The high Fe content of sample 5 is responsible for its higher activity at 300 °C, compared to sample 6. However, this advantage disappears at temperatures of 350 °C or higher, for which temperature sample 6 is significantly more active than sample 5, in spite of the higher Fe content of the latter. Figure 5 summarizes the catalytic results for these samples, while the data are collected in Tables S6–S8 of the Supplementary Materials. In the case of sample 6, the TEM images show intimate contact between Ca^2+^ and metallic Co-Fe NPs. Ca^2+^ exerts a strong influence on the catalytic performance of Co-Fe@C, increasing CO_2_ conversion, and CH_4_ selectivity. It is proposed that Ca^2+^ increases CO_2_ adsorption on Co-Fe@C by forming CaCO_3_, which is the prevalent phase in the material, resulting in an enhanced conversion.

**Figure 6:**
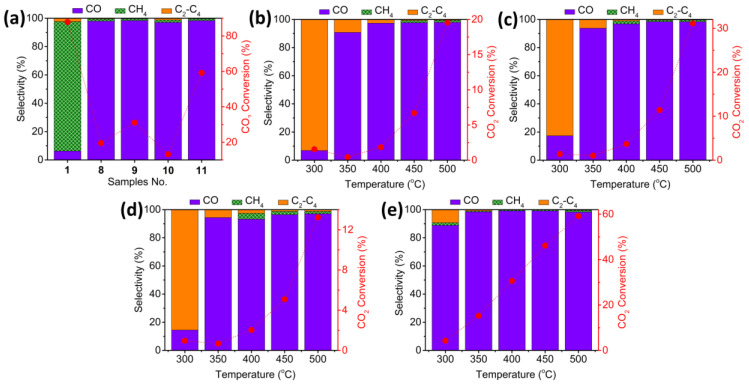
CO_2_ conversion and selectivity for samples 1 and 8–11, with different contents of S (**a**) at 500 °C and (**b**–**e**) at different temperatures. (**b**): Sample 8; (**c**): sample 9; (**d**): sample 10 and (**e**): sample 11. Reaction condition: H_2_/CO_2_ ratio of 7, total flow 4 mL/min, 10 bar, 40 mg catalyst. The error of the measurements is lower than 10%.

Besides the promotion by metallic elements, the effect of S on the catalytic activity of Co-Fe@(N)C was also studied. Samples 8–10 are analogous to sample 1 and were prepared similarly, except that thiourea, as the source of elemental S in three different amounts, was added to the alcogel beads. As shown in Figure 6 and Table S9–S12 in the Supplementary Materials, the presence of S produces two clear effects on the catalytic activity of Co-Fe@(N)C. Firstly, CO_2_ conversion decreases substantially for the three samples 8–10, regardless of the S content, in the range from 2.9 to 7.1 wt % under study. Secondly, the selectivity to CO increases dramatically, being over 97% at the highest temperature tested for these samples. This indicates that S acts as a poison in Co-Fe NPs, diminishing the hydrogenation activity of these NPs. Since CH_4_ formation requires the consumption of four consecutive hydrogen molecules, while CO is formed by the hydrogenation of a single hydrogen molecule, the shift in selectivity from CH_4_ to CO can be easily understood, considering that the less favorable hydrogenation has the higher CO selectivity. Therefore, the poisoning effect of S on Co/Fe catalyst is reflected in the lesser CO_2_ conversion and lesser hydrogen uptake.

An attempt to increase the catalytic activity of the S-containing Co-Fe@(N)C samples was made in sample 11 by adding alkali metal promoters, together with S. It was expected that the basicity introduced by alkali metals could increase the CO_2_ conversion in these S-containing samples, by favoring CO_2_ adsorption. Although the CO_2_ conversion of sample 11 was still lower than that of sample 1, a clear increase in activity attributable to the promotion of Na and K was observed, sample 11 reaching a CO_2_ conversion of 59% at 500 °C, closer to the 88% measured for sample 1, but much higher than the 13% CO_2_ conversion value of sample 10. Notably, the increase in CO_2_ conversion observed for sample 11 did not influence the CO selectivity caused by S poisoning, which, in the case of sample 11, was still over 98%.

## 4. Conclusions

The present study provides catalytic data regarding how the activity and selectivity of Co-Fe@(N)C and Co-Fe@C catalysts can be modulated by promoters. Two classes of effects were observed. Pd in a small percentage and Ca in larger concentrations both increased CO_2_ conversion and CH_4_ selectivity. It is proposed that Pd promotion is due to H_2_ activation and spillover, while Ca enhances CO_2_ adsorption near the sites. On the other hand, S at a few percent decreases activity dramatically, but it does drive selectivity toward CO. It is proposed that S is acting as a poison of the hydrogenating sites, disfavoring not only the attack on CO_2_ but also the successive hydrogen uptake toward CH_4_. The effect of S as a poison is mitigated partially by the basicity of alkali metals. Overall, the present study shows how a range of catalysts, based on Co-Fe and supported on carbon, exhibiting contrasting product selectivity to CH_4_ or to CO, can be prepared by the selection of adequate promoters. Considering the availability of the starting materials and the importance of developing selective CO_2_ hydrogenation processes on a large scale, the present study has shown the way forward for the use of catalysts derived from chitosan/alginate in CO_2_ hydrogenation.

## Figures and Tables

**Table 1:** List of samples under study and their main analytical and physicochemical parameters.

Sample No.	Co (wt %) ^a^	Fe (wt %) ^a^	Promoter (wt %) ^a,b^		C (wt %) ^b^	N (wt %) ^b^	Average Particle Size (nm) ^c^
1	14.1	3.5	-		63.6	1.4	10.5 ± 2
2	18.6	4.4	Pd	0.5	62.9	1.3	10.6 ± 3
3	12.4	3.2	-		70.5	1.6	9.7 ± 5
4	11.3	2.8	Ce	0.4	67.6	1.4	12.2 ± 6 (CeOx): 2.4 ± 1
5	9.0	38.4	-		47.5	-	21.6 ± 5
6	11.3	3.9	Ca	25.8	37.1	-	7.3 ± 3
7	16.0	5.4	Ce	1.5	43.4	-	- ^d^
8	11.7	2.8	S	7.1	49.1	2.2	10.5 ± 2
9	11.3	2.2	S	5.2	53.4	2.5	- ^d^
10	15.6	3.6	S	2.9	55.6	2.7	- ^d^
11	4.3	1.5	K	1.0	60.1	3.9	9.1 ± 6
			Na	1.4			
			S	3.1			

^a^ Determined by ICP-AES analysis after dissolving the metals in aqua regia; ^b^ It is assumed that the rest of the volume up to 100% is the residual oxygen; ^c^ Determined via DF-TEM. ^d^ It was difficult to determine the average particle size from DF-TEM.

## Data Availability

The data presented in this study are available on request from the corresponding author.

## Supplementary Materials

The following supporting information can be downloaded at: https://www.mdpi.com/article/10.3390/nano12183220/s1, Supporting information file includes list of different samples employed in this work, detailed characterization gas chromatography analysis procedure for the catalytic activity, powder XRD patterns of all samples, Raman spectra of samples 2–11, FESEM images of samples 2–11, DF-TEM images for samples 1, 3, 7 and CO_2_ conversion/selectivity data for samples 1-11.
