# Supplementary material for: Promotional Effects on the Catalytic Activity of Co-Fe Alloy Supported on Graphitic Carbon for CO2 Hydrogenation
Source: Nanomaterials (Basel). 2022 Sep 16;12(18):3220. doi: 10.3390/nano12183220 (PMC9506583; doi:10.3390/nano12183220)
Supplement: Supplementary file 1 [file nanomaterials-12-03220-s001.zip › nanomaterials-1857943-supplementary.pdf]

# Supporting Information

# Promotional Effects on the Catalytic Activity of Co-Fe Alloy Supported on Graphitic Carbon for $CO_2$ Hydrogenation

**Bogdan Jurca [1,†], Lu Peng [2,†], Ana Primo [2], Alvaro Gordillo [3], Amarajothi Dhakshinamoorthy [4,5], Vasile I. Parvulescu [1,*] and Hermenegildo García [2,*]**

[1] Department of Organic Chemistry and Biochemistry and Catalysis, Faculty of Chemistry, University of Bucharest, Bdul Regina Elisabeta 4-12, 030016 Bucharest, Romania
[2] Instituto Universitario de Tecnología Química, Universitat Politècnica de València-Consejo Superior de Investigaciones Científicas, Av. De los Naranjos s/n, 46022 Valencia, Spain
[3] BASF SE, 67056 Ludwigshafen am Rhein, Germany; alvaro.gordillo@basf.com
[4] Departamento de Química, Universitat Politècnica de València, Av. De los Naranjos s/n, 46022 Valencia, Spain
[5] School of Chemistry, Madurai Kamaraj University, Madurai 625021, Tamil Nadu, India
* Correspondence: vasile.parvulescu@chemie.unibuc.ro (V.I.P.); hgarcia@qim.upv.es (H.G.)
† The two authors contributed equally.

**Sample characterization**

Field Emission scanning electron microscopy (FESEM) images were acquired by using a JEOL JSM 6300 apparatus. HRTEM images were recorded in a JEOL JEM 2100F under an accelerating voltage of 200 kV. Samples were prepared by applying one drop of the suspended material in ethanol onto a carbon-coated nickel TEM grid and allowing them to dry at room temperature. Raman spectra were collected with a Horiba Jobin Yvon-Labram HR UV-visible-NIR (200-1600 nm) Raman Microscope Spectrometer using a 512 nm laser. The chemical composition of the samples was determined by combustion chemical analysis by using a CHNS FISONS elemental analyser.

**Catalytic Tests**

A setup (Microactivity tester, PID Eng&Tech) equipped with a stainless steel (316 SS) fixed bed tube reactor (Autoclave Engineers) featured with an inner K-type thermocouple was used. Two mass flow controllers (EL-FLOW Select, Bronkhorst) were used to feed the mixture of the inlet gases: hydrogen (5.0, Linde) and carbon dioxide (4.5, Linde). The total gas flow rate was checked before each experiment using a gas calibrated burette connected to the outlet of the reactor setup. After catalyst activation at 300 $^oC$ under $N_2$, an amount of 40 mg catalyst powder was introduced in the reactor. Air was removed by flushing the system at room temperature for 15 min with 30 mL/min $H_2$ and 10 mL/min $CO_2$, followed by 30 min catalytic reaction at the flow rates of different ratio of $H_2$ and $CO_2$ (in total 4 mL/min). Afterwards, the reactor was pressurized at 10 bar depending on different reactions. Each sample was submitted to a 5 h test starting at 250 $^oC$ and increasing the temperature in 50 $^oC$ steps. Each temperature was maintained for 1 h period before increasing another 50 $^oC$. Analysis of the reaction products was carried out on line with multichannel gas chromatography that quantifies the percentage of $CO_2$, CO, $CH_4$ and $C_{2+}$ products. Data at each temperature corresponds to the average value of the analysis measured for each temperature at 30, 45 and 55 min after the stabilization of the temperature. The values of the $CO_2$ conversion obtained from the GC measurements coincided very well in all the experiments, indicating that the reactor setup reached the steady state operation conditions.

GC analyses were performed using $H_2$ as carrier gas on an Agilent 7890A chromatograph equipped with a capillary PLOT column (RT-Msieve 5A, Restek) and a thermal conductivity detector (TCD). Oven temperature program started with a 5 min dwell at 50 $^oC$, then continue with a ramp with 25 $^oC$/min up to 250 $^oC$ followed by a final dwell of

5 min, allowing thus a very good separation between $CH_4$, CO and $CO_2$. The gas samples were injected through a remotely controlled 6-way valve (A4C6WE, Vici) kept at ambient temperature. The reproducibility of the analysis system was checked prior to each experiment by injecting a series of three successive samples of standard gas mixtures of known composition passed through the reactor at room temperature.

**Table S1.** List of samples under study.

| Sample No. | Metal source | m (mg) | Thiourea (mg/mL) |
|---|---|---|---|
| 1 | $CoCl_2$ | 146.5 | - |
| | $FeCl_2$ | 38.9 | |
| 2 | $CoCl_2$ | 146.5 | - |
| | $FeCl_2$ | 38.9 | |
| | $PdCl_2$ | 7.5 | |
| 3 | $Co(OAc)_2$ | 150 | - |
| | $Fe(OAc)_2$ | 50 | |
| 4 | $Co(OAc)_2$ | 150 | - |
| | $Fe(OAc)_2$ | 50 | |
| | $Ce(OAc)_3 \cdot xH_2O$ | 10 | |
| 5 | $CoCl_2 \cdot 6H_2O$ | 150 | - |
| | $FeCl_2$ | 75 | |
| 6 | $Co(OAc)_2$ | 240 | - |
| | $Fe(OAc)_2$ | 100 | |
| 7 | $CoCl_2 \cdot 6H_2O$ | 951.7 | - |
| | $FeCl_2$ | 101.4 | |
| | $Ce(OAc)_3 \cdot xH_2O$ | 126.9 | |
| 8 | $CoCl_2$ | 146.5 | 20 |
| | $FeCl_2$ | 38.9 | |
| 9 | $CoCl_2$ | 146.5 | 5 |
| | $FeCl_2$ | 38.9 | |
| 10 | $CoCl_2$ | 146.5 | 2 |
| | $FeCl_2$ | 38.9 | |
| 11 | $Co(OAc)_2$ | 40 | 2 |
| | $Fe(OAc)_2$ | 16.5 | |
| | NaOAc | 8 | |
| | KOAc | 4 | |

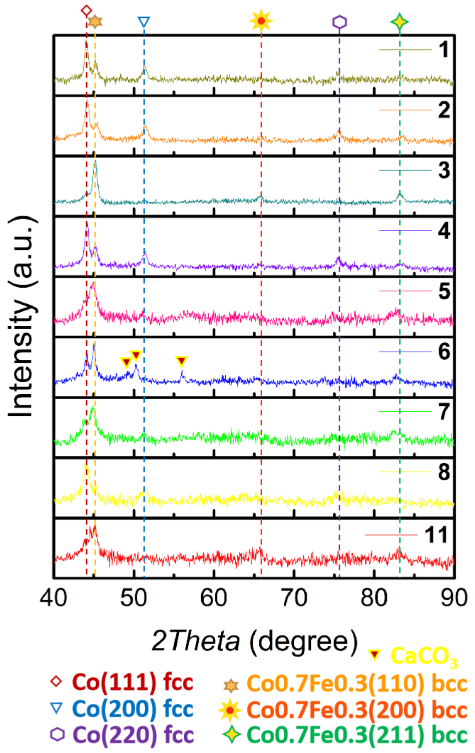


**Figure S1.** XRD patterns of the samples **1**-**11**.

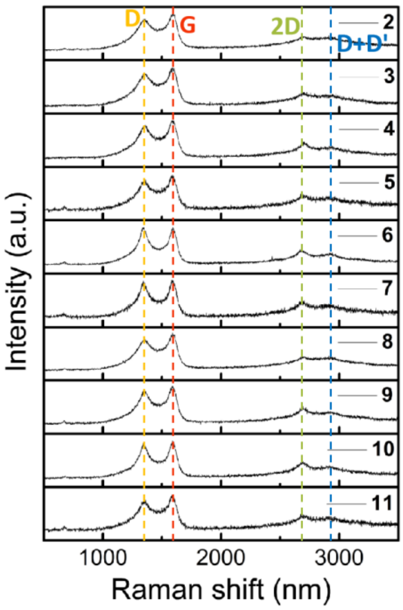


**Figure S2.** Rraman spectrums of the samples 2-11.

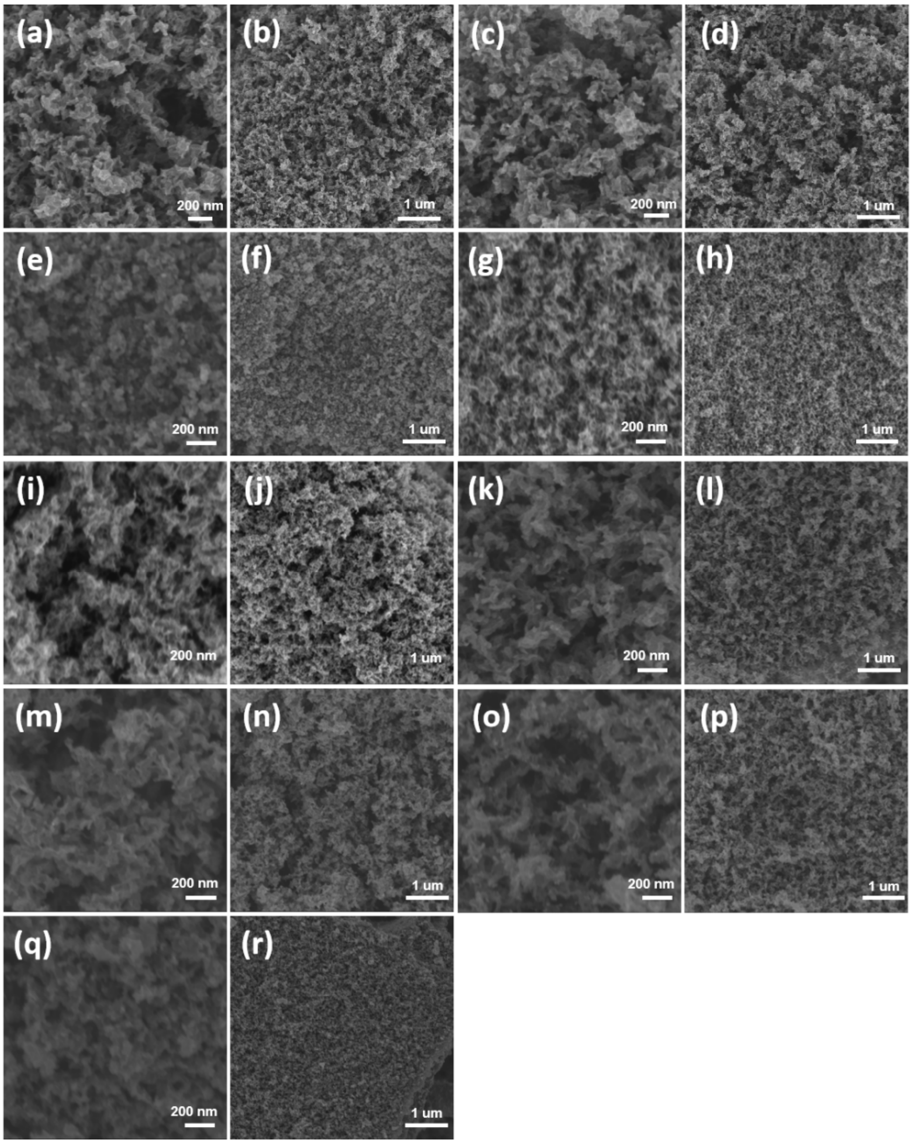


**Figure S3.** FESEM images of samples **2-5**, **7-11**. (**a**, **b**: sample **2**; **c**, **d**: sample **3**; **e**, **f**: sample **4**; **g**, **h**: sample **5**; **i**, **j**: sample **7**; **k**, **l**: sample **8**; **m**, **n**: sample **9**; **o**, **p**: sample **10**; **q**, **r**: sample **11**.).

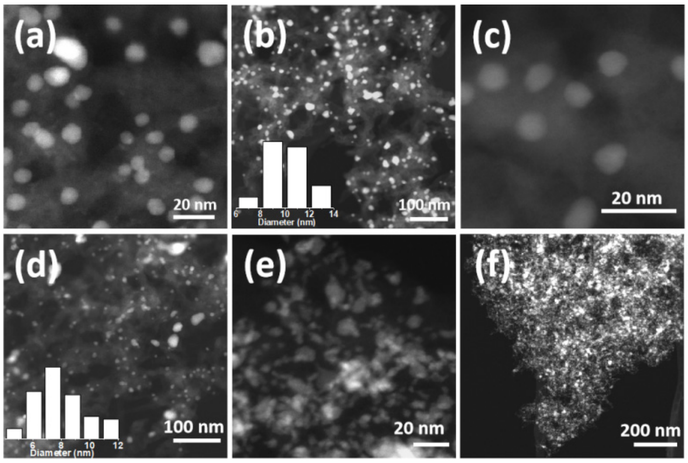


**Figure S4.** DF-TEM images of samples **1**, **3** and **7**. (**a**, **b**: sample **1**; **c**, **d**: sample **3**; **e**, **f**: sample **7**.).

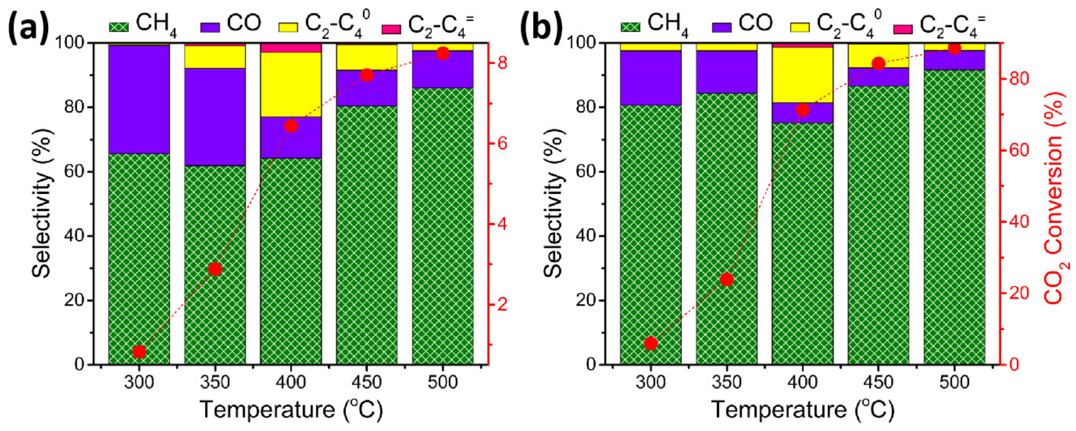


**Figure S5.** $CO_2$ conversion and selectivity for samples **3** and **4** at different temperature. (**a**: sample **3** and **b**: sample **4**. Reaction condition: $H_2/CO_2$ ratio of 7, total flow 4 mL/min, 10 bar, 40 mg catalyst.).

**Table S2.** $CO_2$ conversion and selectivity for sample **1** on different temperature. (Reaction condition: $H_2/CO_2$ ratio of 7, total flow 4 mL/min, 10 bar, 40 mg catalyst.).

| T (°C) | C (%) $CO_2$ | S (%) $CH_4$ | S (%) $C_2H_6$ | S (%) $C_3H_8$ | S (%) *n*-$C_4H_{10}$ | S (%) $C_2H_4$ | S (%) $C_3H_6$ | S (%) CO | S (%) $C_2$-$C_4^0$ | S (%) $C_2$-$C_4^=$ | S (%) $C_2$-$C_4$ |
|---|---|---|---|---|---|---|---|---|---|---|---|
| 300 | 9.8 | 81.3 | 1.3 | 0 | 0 | 0 | 0 | 17.4 | 1.3 | 0 | 1.3 |
| 350 | 32.7 | 76.1 | 4.4 | 1.6 | 1.4 | 0 | 0.5 | 16.0 | 7.4 | 0.5 | 7.9 |
| 400 | 74.4 | 68.4 | 11.9 | 7.4 | 3.5 | 0.6 | 1.4 | 6.9 | 22.8 | 2.0 | 24.8 |
| 450 | 82.9 | 83.0 | 6.9 | 1.8 | 0.6 | 0.3 | 0.3 | 7.2 | 9.3 | 0.5 | 9.8 |
| 500 | 87.9 | 91.4 | 2.1 | 0.2 | 0 | 0.1 | 0 | 6.3 | 2.3 | 0.1 | 2.3 |

**Table S3.** $CO_2$ conversion and selectivity for sample **2** on different temperature. (Reaction condition: $H_2/CO_2$ ratio of 7, total flow 4 mL/min, 10 bar, 40 mg catalyst.).

| T (°C) | C (%) $CO_2$ | S (%) $CH_4$ | S (%) $C_2H_6$ | S (%) $C_3H_8$ | S (%) *n*-$C_4H_{10}$ | S (%) $C_2H_4$ | S (%) $C_3H_6$ | S (%) CO | S (%) $C_2$-$C_4^0$ | S (%) $C_2$-$C_4^=$ | S (%) $C_2$-$C_4$ |
|---|---|---|---|---|---|---|---|---|---|---|---|
| 300 | 19.6 | 68.0 | 6.1 | 2.8 | 3.2 | 0 | 2.1 | 17.9 | 12.1 | 2.1 | 14.1 |
| 350 | 39.6 | 84.4 | 2.4 | 2.3 | 1.6 | 0 | 0.8 | 8.5 | 6.3 | 0.8 | 7.1 |
| 400 | 74.4 | 85.1 | 6.6 | 2.0 | 0.9 | 0 | 0.4 | 5.1 | 9.4 | 0.4 | 9.8 |
| 450 | 83.8 | 89.7 | 4.0 | 0.5 | 0 | 0.1 | 0.1 | 5.5 | 4.6 | 0.3 | 4.8 |
| 500 | 84.7 | 91.4 | 1.5 | 0.2 | 0 | 0 | 0 | 6.9 | 1.7 | 0 | 1.7 |

**Table S4.** $CO_2$ conversion and selectivity for sample **3** on different temperature. (Reaction condition: $H_2/CO_2$ ratio of 7, total flow 4 mL/min, 10 bar, 40 mg catalyst.).

| T (ºC) | C (%) $CO_2$ | S (%) $CH_4$ | S (%) $C_2H_6$ | S (%) $C_3H_8$ | S (%) $n$-$C_4H_{10}$ | S (%) $C_2H_4$ | S (%) $C_3H_6$ | S (%) CO | S (%) $C_2$-$C_4^0$ | S (%) $C_2$-$C_4^=$ | S (%) $C_2$-$C_4$ |
|---|---|---|---|---|---|---|---|---|---|---|---|
| 300 | 8.2 | 65.6 | 0.7 | 0 | 0 | 0 | 0 | 33.7 | 0.7 | 0 | 0.7 |
| 350 | 28.8 | 61.9 | 3.8 | 1.6 | 1.6 | 0 | 0.9 | 30.3 | 7.0 | 0.9 | 7.9 |
| 400 | 64.4 | 64.2 | 11.4 | 5.3 | 3.4 | 0.8 | 2.1 | 12.8 | 20.1 | 2.9 | 23.0 |
| 450 | 77.0 | 80.3 | 6.3 | 1.3 | 0.4 | 0.2 | 0.3 | 11.2 | 8.0 | 0.5 | 8.5 |
| 500 | 82.4 | 86.0 | 2.2 | 0.2 | 0 | 0 | 0 | 11.6 | 2.4 | 0 | 2.4 |

**Table S5.** $CO_2$ conversion and selectivity for sample **4** on different temperature. (Reaction condition: $H_2/CO_2$ ratio of 7, total flow 4 mL/min, 10 bar, 40 mg catalyst.).

| T (ºC) | C (%) $CO_2$ | S (%) $CH_4$ | S (%) $C_2H_6$ | S (%) $C_3H_8$ | S (%) $n$-$C_4H_{10}$ | S (%) $C_2H_4$ | S (%) $C_3H_6$ | S (%) CO | S (%) $C_2$-$C_4^0$ | S (%) $C_2$-$C_4^=$ | S (%) $C_2$-$C_4$ |
|---|---|---|---|---|---|---|---|---|---|---|---|
| 300 | 6.0 | 80.6 | 2.5 | 0 | 0 | 0 | 0 | 16.9 | 2.5 | 0 | 2.5 |
| 350 | 23.9 | 84.4 | 2.4 | 0 | 0 | 0 | 0 | 13.2 | 2.4 | 0 | 2.4 |
| 400 | 71.3 | 75.2 | 9.8 | 5.2 | 2.2 | 0.3 | 1.1 | 6.2 | 17.2 | 1.4 | 18.6 |
| 450 | 84.3 | 86.6 | 5.8 | 1.3 | 0.3 | 0.2 | 0.2 | 5.6 | 7.4 | 0.4 | 7.8 |
| 500 | 88.6 | 91.6 | 2.2 | 0.3 | 0 | 0 | 0 | 6.0 | 2.4 | 0 | 2.4 |

**Table S6.** $CO_2$ conversion and selectivity for sample **5** on different temperature. (Reaction condition: $H_2/CO_2$ ratio of 7, total flow 4 mL/min, 10 bar, 40 mg catalyst.).

| T (ºC) | C (%) $CO_2$ | S (%) $CH_4$ | S (%) $C_2H_6$ | S (%) $C_3H_8$ | S (%) $n$-$C_4H_{10}$ | S (%) $C_2H_4$ | S (%) $C_3H_6$ | S (%) CO | S (%) $C_2$-$C_4^0$ | S (%) $C_2$-$C_4^=$ | S (%) $C_2$-$C_4$ |
|---|---|---|---|---|---|---|---|---|---|---|---|
| 250 | 6.8 | 86.0 | 3.0 | 7.6 | 0 | 0 | 0 | 3.4 | 10.6 | 0 | 10.6 |
| 300 | 24.5 | 92.0 | 3.6 | 1.5 | 0 | 0 | 0 | 2.9 | 5.1 | 0 | 5.1 |
| 350 | 50.9 | 88.4 | 6.6 | 2.0 | 0.8 | 0 | 0 | 2.3 | 9.3 | 0 | 9.3 |
| 400 | 72.2 | 90.2 | 6.2 | 1.1 | 0.3 | 0 | 0 | 2.2 | 7.5 | 0 | 7.5 |
| 450 | 80 | 94.5 | 2.7 | 0.1 | 0 | 0 | 0 | 2.7 | 2.8 | 0 | 2.8 |

**Table S7.** $CO_2$ conversion and selectivity for sample **6** on different temperature. (Reaction condition: $H_2/CO_2$ ratio of 7, total flow 4 mL/min, 10 bar, 40 mg catalyst.).

| T (ºC) | C (%) $CO_2$ | S (%) $CH_4$ | S (%) $C_2H_6$ | S (%) $C_3H_8$ | S (%) $n$-$C_4H_{10}$ | S (%) $C_2H_4$ | S (%) $C_3H_6$ | S (%) CO | S (%) $C_2$-$C_4^0$ | S (%) $C_2$-$C_4^=$ | S (%) $C_2$-$C_4$ |
|---|---|---|---|---|---|---|---|---|---|---|---|
| 300 | 32.1 | 95.8 | 1.8 | 0.8 | 0 | 0 | 0 | 1.7 | 2.5 | 0 | 2.5 |
| 350 | 65.6 | 95.9 | 2.3 | 0.3 | 0 | 0 | 0 | 1.5 | 2.6 | 0 | 2.6 |
| 400 | 84.8 | 96.9 | 1.6 | 0.1 | 0 | 0 | 0 | 1.4 | 1.7 | 0 | 1.7 |
| 450 | 92.1 | 97.9 | 0.8 | 0.1 | 0 | 0 | 0 | 1.2 | 0.9 | 0 | 0.9 |

| | | | | | | | | | | | |
|---|---|---|---|---|---|---|---|---|---|---|---|
| 500 | 94.2 | 97.8 | 0.6 | 0.1 | 0 | 0 | 0 | 1.5 | 0.7 | 0 | 0.7 |

**Table S8.** $CO_2$ conversion and selectivity for sample **7** on different temperature. (Reaction condition: $H_2/CO_2$ ratio of 4, total flow 4 mL/min, 10 bar, 40 mg catalyst.).

| T (°C) | C (%) $CO_2$ | S (%) $CH_4$ | S (%) $C_2H_6$ | S (%) $C_3H_8$ | S (%) $n$-$C_4H_{10}$ | S (%) $C_2H_4$ | S (%) $C_3H_6$ | S (%) CO | S (%) $C_2$-$C_4^0$ | S (%) $C_2$-$C_4^=$ | S (%) $C_2$-$C_4$ |
|---|---|---|---|---|---|---|---|---|---|---|---|
| 250 | 1.4 | 41.4 | 16.0 | 17.8 | 0 | 0 | 0 | 24.8 | 33.8 | 0 | 33.8 |
| 300 | 4.8 | 54.4 | 3.7 | 8.8 | 0 | 0 | 0 | 33.1 | 12.5 | 0 | 12.5 |
| 350 | 12.3 | 49.9 | 5.3 | 2.2 | 2.7 | 0 | 0 | 39.8 | 10.3 | 0 | 10.3 |
| 400 | 37.3 | 61.3 | 10.5 | 5.1 | 1.9 | 0 | 0.3 | 20.9 | 17.5 | 0.3 | 17.8 |
| 450 | 62.0 | 79.2 | 7.6 | 1.4 | 0.2 | 0 | 0.1 | 11.5 | 9.1 | 0.1 | 9.2 |

**Table S9.** $CO_2$ conversion and selectivity for sample **8** on different temperature. (Reaction condition: $H_2/CO_2$ ratio of 3, total flow 4 mL/min, 10 bar, 40 mg catalyst.).

| T (°C) | C (%) $CO_2$ | S (%) $CH_4$ | S (%) $C_2H_6$ | S (%) $C_3H_8$ | S (%) $n$-$C_4H_{10}$ | S (%) $C_2H_4$ | S (%) $C_3H_6$ | S (%) CO | S (%) $C_2$-$C_4^0$ | S (%) $C_2$-$C_4^=$ | S (%) $C_2$-$C_4$ |
|---|---|---|---|---|---|---|---|---|---|---|---|
| 300 | 1.6 | 0 | 14.4 | 48.1 | 0 | 0 | 30.7 | 6.9 | 62.5 | 30.7 | 93.1 |
| 350 | 0.5 | 0 | 9.2 | 0 | 0 | 0 | 0 | 90.8 | 9.2 | 0 | 9.2 |
| 400 | 1.8 | 0 | 2.7 | 0 | 0 | 0 | 0 | 97.3 | 2.7 | 0 | 2.7 |
| 450 | 6.7 | 1.6 | 0.8 | 0 | 0 | 0 | 0 | 97.7 | 0.8 | 0 | 0.8 |
| 500 | 19.6 | 1.5 | 0.6 | 0 | 0 | 0 | 0 | 98.0 | 0.6 | 0 | 0.6 |

**Table S10.** $CO_2$ conversion and selectivity for sample **9** on different temperature. (Reaction condition: $H_2/CO_2$ ratio of 2, total flow 4 mL/min, 10 bar, 40 mg catalyst.).

| T (°C) | C (%) $CO_2$ | S (%) $CH_4$ | S (%) $C_2H_6$ | S (%) $C_3H_8$ | S (%) $n$-$C_4H_{10}$ | S (%) $C_2H_4$ | S (%) $C_3H_6$ | S (%) CO | S (%) $C_2$-$C_4^0$ | S (%) $C_2$-$C_4^=$ | S (%) $C_2$-$C_4$ |
|---|---|---|---|---|---|---|---|---|---|---|---|
| 300 | 1.4 | 0 | 19.8 | 40.9 | 0 | 0 | 21.9 | 17.4 | 60.7 | 21.9 | 82.7 |
| 350 | 1.0 | 0 | 6.2 | 0 | 0 | 0 | 0 | 93.8 | 6.2 | 0 | 6.2 |
| 400 | 3.7 | 2.1 | 1.1 | 0 | 0 | 0 | 0 | 96.9 | 1.1 | 0 | 1.1 |
| 450 | 11.4 | 1.1 | 0.5 | 0 | 0 | 0 | 0 | 98.4 | 0.5 | 0 | 0.5 |
| 500 | 31.1 | 1.2 | 0.4 | 0 | 0 | 0 | 0 | 98.4 | 0.4 | 0 | 0.4 |

**Table S11.** $CO_2$ conversion and selectivity for sample **10** on different temperature. (Reaction condition: $H_2/CO_2$ ratio of 1, total flow 4 mL/min, 10 bar, 40 mg catalyst.).

| T (°C) | C (%) $CO_2$ | S (%) $CH_4$ | S (%) $C_2H_6$ | S (%) $C_3H_8$ | S (%) $n$-$C_4H_{10}$ | S (%) $C_2H_4$ | S (%) $C_3H_6$ | S (%) CO | S (%) $C_2$-$C_4^0$ | S (%) $C_2$-$C_4^=$ | S (%) $C_2$-$C_4$ |
|---|---|---|---|---|---|---|---|---|---|---|---|
| 300 | 1.0 | 0 | 23.3 | 38.7 | 0 | 0 | 23.5 | 14.5 | 62.0 | 23.5 | 85.5 |
| 350 | 0.7 | 0 | 5.6 | 0 | 0 | 0 | 0 | 94.4 | 5.6 | 0 | 5.6 |
| 400 | 2.0 | 4.2 | 2.6 | 0 | 0 | 0 | 0 | 93.2 | 2.6 | 0 | 2.6 |
| 450 | 5.1 | 2.3 | 1.2 | 0 | 0 | 0 | 0 | 96.5 | 1.2 | 0 | 1.2 |
| 500 | 13.2 | 1.7 | 1.2 | 0 | 0 | 0 | 0 | 97.1 | 1.2 | 0 | 1.2 |

**Table S12.** $CO_2$ conversion and selectivity for sample **11** on different temperature. (Reaction condition: $H_2/CO_2$ ratio of 1, total flow 4 mL/min, 10 bar, 40 mg catalyst.).

| T (°C) | C (%) $CO_2$ | S (%) $CH_4$ | S (%) $C_2H_6$ | S (%) $C_3H_8$ | S (%) $n$-$C_4H_{10}$ | S (%) $C_2H_4$ | S (%) $C_3H_6$ | S (%) CO | S (%) $C_2$-$C_4^0$ | S (%) $C_2$-$C_4^=$ | S (%) $C_2$-$C_4$ |
|---|---|---|---|---|---|---|---|---|---|---|---|
| 300 | 4.3 | 1.8 | 3.4 | 0 | 0 | 0 | 5.9 | 88.9 | 3.4 | 5.9 | 9.3 |
| 350 | 15.3 | 0.6 | 0.9 | 0 | 0 | 0 | 0 | 98.5 | 0.9 | 0 | 0.9 |
| 400 | 30.6 | 0.6 | 0.3 | 0 | 0 | 0 | 0 | 99.1 | 0.3 | 0 | 0.3 |
| 450 | 46.2 | 0.7 | 0.2 | 0 | 0 | 0 | 0 | 99.1 | 0.2 | 0 | 0.2 |
| 500 | 59.2 | 1.2 | 0.5 | 0 | 0 | 0 | 0 | 98.3 | 0.5 | 0 | 0.5 |
